# Normal pressure hydrocephalus: Diagnostic and predictive evaluationon

**DOI:** 10.1590/S1980-57642009DN30100003

**Published:** 2009

**Authors:** Benito Pereira Damasceno

**Affiliations:** Unidade de Neuropsicologia e Neurolinguística, Departamento de Neurologia, Faculdade de Ciências Médicas, Universidade Estadual de Campinas, SP, Brazil.

**Keywords:** normal pressure hydrocephalus, neuropsychological tests, cerebrospinal fluid tap test, shunt surgery

## Abstract

In typical cases, normal pressure hydrocephalus (NPH) manifests itself with the triad of gait disturbance, which begins first, followed by mental deterioration and urinary incontinence associated with ventriculomegaly (on CT or MRI) and normal cerebrospinal fluid (CSF) pressure. These cases present minor diagnostic difficulties and are the most likely to improve after shunting. Problems arise when NPH shows atypical or incomplete clinical manifestations (25–50% of cases) or is mimicked by other diseases. In this scenario, other complementary tests have to be used, preferentially those that can best predict surgical outcome. Radionuclide cisternography, intracranial pressure monitoring (ICP) and lumbar infusion tests can show CSF dynamics malfunction, but none are able to confirm whether the patient will benefit from surgery. The CSF tap test (CSF-TT) is the only procedure that can temporarily simulate the effect of definitive shunt. Since the one tap CSF-TT has low sensitivity, it cannot be used to exclude patients from surgery. In such cases, we have to resort to a repeated CSF-TT (RTT) or continuous lumbar external drainage (LED). The most reliable prediction would be achieved if RTT or LED proved positive, in addition to the occurrence of B-waves during more than 50% of ICP recording time. This review was based on a PubMed literature search from 1966 to date. It focuses on clinical presentation, neuroimaging, complementary prognostic tests, and differential diagnosis of NPH, particularly on the problem of selecting appropriate candidates for shunt.

## Abstract (pt)

Em casos típicos, a hidrocefalia de pressão normal (HPN) manifesta-se com a tríade: distúrbio da marcha, que começa primeiro, seguido de deterioração mental e incontinência urinária associados a ventriculomegalia (na TC ou RM) e pressão liquórica normal. Esses casos conferem pouca dificuldade diagnóstica e são os que mais provavelmente melhoram após a derivação liquórica. O problema é quando a HPN manifesta-se de forma incompleta ou atípica (25–50% dos casos) ou é mimetizada por outras doenças. Então, outros testes complementares têm que ser usados, preferencialmente aqueles que melhor predizem o resultado cirúrgico. A cisternocintilografia, o monitoramento da pressão intracraniana e o teste de infusão lombar podem realmente mostrar disfunção da dinâmica liquórica, mas nenhum deles pode confirmar se o paciente vai beneficiar-se da cirurgia. O teste de punção liquórica é o único que pode temporariamente simular o efeito definitivo da derivação. Uma vez que o teste de (uma única) punção liquórica tem baixa sensibilidade, ele não pode ser usado para excluir pacientes da cirurgia. Em tais casos, temos que apelar para o teste da punção liquórica repetida (PLR) ou da drenagem liquórica lombar externa contínua (DLE). Um diagnóstico preditivo mais seguro seria conseguido se a PLR ou a DLE é positiva, juntamente com a ocorrência de ondas B em mais que 50% do tempo de monitoramento da pressão intracraniana. Este artigo de revisão baseou-se em uma busca na literatura, via PubMed, desde 1966. Ele focaliza a apresentação clínica, diagnóstico diferencial, testes prognósticos complementares e a questão da seleção de candidatos apropriados para a derivação.

Normal pressure hydrocephalus (NPH) is characterized by the triad of gait disturbance, progressive mental deterioration and urinary incontinence associated with enlargement of the ventricular system and normal cerebrospinal fluid (CSF) pressure. In NPH, the CSF pressure may be normal at one spinal tap, but episodes of increased CSF pressure do occur in this syndrome. For this reason, other terms such as “intermittent pressure hydrocephalus”, “adult hydrocephalus syndrome”, and “adult symptomatic hydrocephalus” could be deemed more appropriate.

NPH has been regarded as a rare cause of dementia with estimates ranging from 0% to 5% of all demented patients. This variable rate depends on whether the diagnosis is based solely on clinical and neuroimaging data or on improvement after a shunt (shunt-responsive NPH). Epidemiological data on NPH incidence and prevalence are scarce, but recent surveys in Germany and Norway^1,2^ have estimated the annual incidence of idiopathic NPH to be between 1.8/100,000 and 5.5/100,000 inhabitants, with a prevalence of 22/100,000.

## Pathophysiology

The CSF is normally produced in the choroid plexus within the lateral and fourth ventricles, from which it passes through the foramina of Luschka and Magendie and enters the cisterna magna, then bathing the superior cerebral convexities in the subarachnoid space, and finally being absorbed by the arachnoid granulations, mainly in the superior sagittal sinus. In hydrocephalus (Gk. *hydro*-, water + *cephalus*, head), the excessive accumulation of CSF can be due to an obstruction into the brain ventricles (“noncommunicating” or “obstructive”, for example by aqueduct stenosis) or to an impairment of CSF flow distally to the fourth ventricle (“communicating”), almost always at the level of the basal cisterns, as most often happens in NPH. In about 50% of patients with communicating NPH there is a known cause (“secondary NPH”, or SNPH), such as meningitis, subarachnoid hemorrhage, or cranial trauma, while the other 50% of cases are idiopathic (INPH).^3^ SNPH may present at any age, while INPH usually presents in the 6^th^ or 7^th^ decade of life.^4^

In INPH, clinical deterioration is probably due to impaired periventricular blood flow associated to interstitial edema, ependyma disruption, microvascular infarctions, gliosis, and neuronal degeneration.^5^ Neuronal injury may result from mechanical stretching of periventricular tissue by the enlarging ventricles, impairment of blood brain barrier, reduced CSF turnover and disturbed elimination of neurotoxic substances such as β-amyloid, tau-protein, and pro-inflammatory cytokines.^6,7^ This decreased CSF clearance may also explain the high co-occurrence of Alzheimer-like changes in the cortex of INPH patients and of rats with experimental chronic hydrocephalus^8,9^. A possible association with Alzheimer’s disease, particularly when there is concurrent arterial hypertension and cerebral arteriosclerosis, may explain why many NPH cases remain with severe cognitive and motor deficits after shunting, even when ventricular size decreases postoperatively.^3,7^

## Clinical symptoms and signs

The complete triad is seen in 50–75% of cases, with gait and cognitive disturbances occurring in 80–95%, and urinary incontinence in 50–75% of cases.^10^ In typical cases, gait disturbance is the first and most salient sign, followed by forgetfulness or mild dementia, psychomotor retardation, apathy (with parkinsonian or depressive appearance) and, later on, urinary urgency or incontinence. These cases present minor diagnostic difficulties and they are the most likely to improve after shunting.^11-13^

## Gait disturbance

In fully developed INPH, the cardinal sign is a broad-based, short-step, magnetic gait with start hesitation and increased instability on turning, often with falls. In mild cases the gait may be merely ataxic and wide-based. Although INPH gait shares the features of gait in Parkinson disease, progressive supranuclear palsy, and cerebellar ataxia, the nature of INPH gait is closest to apraxia of gait, which may be explained by gait ignition failure, probably caused by frontal dysfunction.^14^ Nevertheless, the term “gait apraxia” has been considered inappropriate in INPH, since these patients may execute walking movements without difficulty when supported or lying down, in spite of freezing their gait as soon as they try to start walking.^15^ Therefore, this gait disturbance has also been explained by a disconnection between the frontal cortex and the basal ganglia, uninhibited antigravity reflexes, and co-contraction of agonists and antagonists during walking.^15,16^ This explanation is in line with findings of reduced blood flow and oxygen metabolism in the basal ganglia as well as in the frontal lobe, thalamus, hippocampus, and periventricular white matter.^17,18^

Additional motor signs are commonly found at examination: postural instability with tendency to fall backwards (due to impaired postural reflexes), spastic paraparesis, hyper-reflexia, paratonic rigidity, and primitive reflexes such as snouting, palmomental, sucking, grasping, and Babinski sign.^19^

### Cognitive deficit and astheno-emotional syndrome

The cognitive impairment is typically of the “subcortical” type, with inattention, memory impairment, psychomotor slowing, apathy, and difficulty with executive functions. Unlike Alzheimer’s disease, “cortical” signs such as apraxia, agnosia and aphasia are rare; and delayed memory recall is severely impaired, while delayed recognition is less affected or even normal. INPH dementia syndrome is usually mild, and when it is the preceding or predominant clinical sign, particularly if severe, then the most probable diagnosis is Alzheimer’s disease and not NPH. The cognitive deficits are often accompanied by other mental symptoms which constitute an astheno-emotional syndrome (concentration difficulties, increased fatigability, irritability, emotional instability, and in most severe cases, emotional and motivational blunting), sometimes with impaired wakefulness and mental confusion.^20,21^

### Urinary incontinence

Usually presents thirdly after gait and cognitive disorder, but at early stages urinary frequency and urgency may be present. These symptoms are due to stretching of periventricular nerve fibers with subsequent loss of voluntary supraspinal control (uninhibited) of bladder contractions.^22^ Detrusor overactivity mostly underlies urinary urgency/frequency and incontinence in INPH.^23^ As the disease progresses, even fecal incontinence may occur.

## Diagnosis

The diagnosis of NPH is usually based on the following criteria:

(1) a history of gait disturbance, progressive mental deterioration, and urinary urgency or incontinence;
(2) hydrocephalus, defined as Evans’ ratio (the ratio between the maximal width of the frontal horns and the internal diameter of the skull at the same level) above 0.30 on computerized tomography (CT) or magnetic resonance (MR) image; and
(3) a mean CSF pressure below 18 or 20 cm of water.

Particularly for research purposes, INPH can be classified according to the 2005 consensus guidelines^24^ into “probable”, “possible” or “unlikely” on the basis of history, clinical findings, brain imaging data, and CSF opening pressure. Even if we adhere to these criteria, which can yield a 65% positive predictive value and an 82% negative predictive value,^25^ we cannot definitively rule out other dementing conditions. Therefore, the “gold standard” for diagnosis remains clinical improvement after CSF shunting. In the Japanese guidelines for INPH,^26^ surgical indication should be determined by the clinical symptoms, MRI findings, and CSF tap test, with the diagnosis of “probable” INPH being based on improved gait after the CSF tap test or continuous CSF drainage, whereas the diagnosis of “definite” INPH is based on improvement of symptoms after shunt surgery.

## Differential diagnosis

Differential diagnostic problems may arise in some patients with atypical or incomplete clinical manifestations, as well as in patients with “subcortical” dementia (subcortical arteriosclerotic encephalopathy or Binswanger’s disease, progressive supranuclear palsy, diffuse Lewy body disease, Parkinson’s disease), which can mimic the clinical picture of INPH. Subcortical arteriosclerotic encephalopathy is in fact much more common than INPH, and should be included in the differential diagnosis as the most probable cause of the “classical” triad.^20^ INPH may sometimes mimic Parkinson’s disease,^28^ but INPH can be distinguished by its more broad-based, wider outward rotation of the feet, diminished step height, relatively preserved arm swing, and more erect trunk.^29^ In elderly patients, other more common conditions than these can also explain gait difficulties (e.g., peripheral neuropathy, cervical or lumbar stenosis, arthritis, vestibular diseases) and urinary incontinence (prostate disease, stress incontinence, chronic urinary tract infection). Differentiation from Alzheimer’s disease (AD) is rarely difficult, since in AD the dementia precedes and predominates over the motor and urinary symptoms, which typically manifest later on, often years afterwards; while in AD “cortical” symptoms prevail such as aphasia, agnosia, apraxia, as well as amnesia with impaired recognition (hippocampal amnesia).

## Diagnostic and prognostic supplementary tests

The limitations and uncertainties associated with the clinical diagnosis of NPH have stimulated the search for more accurate methods and criteria for selection of patients for shunt surgery, which can benefit 25–80% of these patients but has complication rates (35–52%) that dissuade us from shunting every case of suspected NPH.^12-13^

### Neuropsychological tests

The testing should cover the cognitive areas most impaired in INPH: attention, speed of information processing, working memory, executive functions, and memory (both delayed recall and recognition of series of words and pictures).^30-32^

### Neuroimaging

In typical cases of INPH, computerized tomography (CT) and magnetic resonance imaging (MRI) show ventricular enlargement out of proportion to cerebral atrophy, with associated ballooning of frontal horns, periventricular hyperintensities, thinning and elevation of the corpus callosum, and widening of temporal horns without evidence of hippocampal atrophy. The presence of periventricular hyperintensity and associated subcortical lacunar infarctions in NPH does not predict poor surgical outcome and should not exclude patients from shunting.^33^ By the same token, the presence of enlarged basal cisterns and Sylvian fissures and of focally dilated sulci tend to support rather than exclude the diagnosis of shunt-responsive INPH.^34^ In normal aging and degenerative processes such as Alzheimer’s and Pick’s disease, on the contrary, the thinning of the gyri and dilation of the sulci are more generalized, occurring to a similar degree in the affected brain regions.^35^

The results of CT, MRI or radionuclide cisternography can be inconclusive and insufficient to establish a correct diagnosis and particularly to predict which patients will improve after shunt surgery. A “positive” radionuclide cisternography (with ventricular reflux and convexity block) is not specific for NPH and can be seen in other dementia disorders and even in healthy subjects.^36^ The predictive value of radionuclide cisternography has been repeatedly questioned, such that Vanneste et al.^37^ suggested it should no longer be performed as it will not reduce the diagnostic uncertainty remaining after clinical and CT evaluation. Even MRI (with CSF voiding sign in the aqueduct) has been criticized for its questionable additional predictive value, besides its limited availability and high costs. Some studies^38,39^ have shown correlation between the increase of CSF flow in the aqueduct (“void sign”) and shunt results, while in other studies^40^ the correlation was low (statistically not significant), with the same frequency of void sign occurring in the groups of NPH patients and healthy controls. More recent studies^41^ have shown that the measurement of peak CSF flow velocity at the level of the aqueduct, before and after lumbar CSF drainage, by using cine phase-contrast MRI, is a sensitive method to support the diagnosis of NPH and to select patients who are likely to benefit (or not) from shunt surgery.

Single photon emission computerized tomography (SPECT) and positron emission tomography (PET) can show reduction of cerebral blood flow and metabolism, mainly in frontobasal and anterior periventricular regions, but their diagnostic and prognostic value is not well established and they are not part of the routine selection procedures for shunt surgery.

### Intracranial pressure (ICP) monitoring

The continuous long-term monitoring of ICP commonly shows elevations of CSF pressure (so called B waves), which have long been regarded as highly predictive of good postsurgical outcome, particularly when they occur during more than 50% of ICP recording time.^3,10^

### Lumbar CSF infusion test

The infusion of saline or artificial CSF into the ventricle or lumbar subarachnoid space raises the resistance to CSF outflow with subsequent increase of CSF pressure, which in NPH patients reaches higher levels than the plateau observed in normal individuals. Most investigators agree that a resistance to CSF flow of 18 mm Hg/ml per minute or higher, predicts good surgical outcome in these patients.^42,43^ More recent studies^2^ have found that, among the infusion-derived parameters, CSF pressure pulsatility rather than resistance to CSF outflow, is linked to shunt response.

The problem with CSF infusion tests is that their reliability depends on high technical expertise, which may not be available in many neurosurgical services. Furthermore, as regards ICP monitoring, the interpretation of the recorded pressure oscillations has not followed standardized criteria, and B-wave frequency, amplitude and morphology vary according to the different sleep stages and have been recorded even in non-hydrocephalic persons.^44^ In spite of the positive findings pertaining to both methods, other authors have questioned their predictive value.^45-48^

### CSF tap test

Adams et al.^49^ and Fisher^50^ originally described the beneficial (though transient) effect of CSF removal in patients with hydrocephalic dementia, as well as the improvement of these patients after shunt surgery. This method was later improved by Wikkelsö et al.^51,52^ and others^53,54^ by introducing the quantitative testing of gait and cognitive functions before and after the drainage of 40–50 ml lumbar CSF (CSF tap test or CSF-TT). These authors found that CSF-TT can predict not only the outcome of surgery but also the degree of improvement.

Lately, however, this test has been criticized for its high rate of false negatives, which has led some authors to introduce the continuous lumbar external drainage (LED)^26,55^ or the repeated lumbar CSF tap test (RTT).^56^ In these studies, RTT taps were performed on three consecutive days and at each tap a minimum of 30 to 40 ml of CSF was removed, while the LED was performed continuously for 3 to 5 days, with a minimum of 150 ml of CSF drained daily. In fact, with the introduction of these two procedures the (one tap) CSF-TT sensitivity (26–61%) and positive predictive value (73–100%) was improved upon, with LED showing a sensitivity of 50–100%, specificity of 60–100%, and positive predictive value of 80–100%.^43^ Although these procedures could improve the accuracy of CSF-TT, they can have higher complication rates (meningitis, nerve root inflammation, subdural hematoma), besides requiring hospitalization, with higher costs and greater suffering for the patient.

At the UNICAMP hospital, in Campinas, Brazil, we have attempted to improve the predictive value of the one tap version of CSF-TT since 1988 by increasing the amount of CSF removed and the drainage duration.^19,30,57,58^ The selection of subtests takes into account that they should (1) be sensitive to NPH motor manifestations and measure cognitive functions that usually improve after LP and shunting; (2) be suited even for illiterate subjects, on account of the high illiteracy rate of our population of patients (about 15%); and (3) be low cost and easy and rapid to administer by neurologists in ambulatory outpatients. In order to rule out other diseases, we carried out a comprehensive investigation including CT or MRI, radionuclide cisternography, CSF analysis, laboratory tests, the Mini-Mental State Examination,^59,60^ CAMDEX,^61^ and use of Hachinski Ischemic Scores for vascular dementia,^62^ ICD-10^63^ and NINCDS-ADRDA criteria for Alzheimer’s disease,^64^ besides gait and memory tests.

With the first version of our CSF-TT (used from 1988 to 1995)^19^, 50 ml of CSF was removed, and there was good correlation between shunting results (*phi* coefficient=0.48, *p*<0.05) and the gait and memory tests [the tests for visuo-motor speed (cylinders test) and visuo-construction showed no correlation].

In the second CSF-TT version (in use since 1996),^58^ 50 ml to 100 ml of CSF were removed, the tests for visuo-motor speed and visuo-constructive skills were excluded, while the gait and memory tests were maintained and tests for postural reaction were added. Twenty-eight (78%) of the 36 NPH operated patients improved. Eleven (30%) had postsurgical complications (mainly subdural hematoma). The results of this second version were correlated to those of shunt surgery (*p*<0.01), particularly as regards gait test (*r*=1, *p*<0.001), and its additional predictive value was 24% compared to the predictive value of clinical and tomographic data alone.

The second version of our CSF-TT was performed in two consecutive days and at the same time each day. In the morning of the first day (about 11 o’clock) the patients underwent testing of memory and gait. On the second day, early in the morning (about eight o’clock), CSF was drained by lumbar puncture (LP) for three hours (until eleven o’clock) or until a maximum of 100 ml CSF was removed. The best score obtained by the patient on each subtest out of these two post-LP evaluations was considered their post-LP score. Postsurgical follow-up examination (with neuropsychological and gait tests) was performed at 3 and 6 months, at 1 year, and yearly thereafter. CT was repeated at the 6^th^ month follow-up. If the patient had not improved, we examined the shunt function by percutaneously testing the proximal and distal patency of the shunt (and the valve function), as well as by analyzing changes in ventricular size on CT scan.

In the *gait test*, the examiner recorded (with a chronometer) the time taken by the patient to walk 18 meters as quickly as possible. The mean value of four attempts was the patient’s gait score. In the memory (*verbal learning*) test, the subject was given ten trials to learn a list of 10 unrelated words presented orally by the examiner (water, flower, cat, key, stone, cross, street, cake, hand, wind). The patient’s score (maximum of 10) was the mean of summed total recall across the ten acquisition trials. At the post-LP memory testing, the words presented before LP were replaced by another 10 words (house, ox, bread, night, bell, light, bridge, table, foot, rain) in order to avoid any learning effect. Improvement in the gait test was defined as an increase of at least 5%, and in the memory test as an increase of at least 20%. CSF-TT was considered positive if the patient’s performance improved in the two tests or only in the gait test.

### Surgical treatment

As yet the only efficacious treatment for NPH has been surgical diversion of CSF. Other alternatives such as acetazolamide (250 mg to 500 mg daily) and repeated lumbar punctures can yield mild and transient relief of symptoms, and are justified in patients with high surgical risks.^65^ The surgical procedure is usually a ventriculoperitoneal shunt (VPS), placing the proximal catheter within the ventricles through the right hemisphere posterior parietal region, and the distal catheter into the peritoneal cavity. In cases with previous peritonitis or abdominal surgeries, a ventriculoatrial shunt is the alternative. The VPS may use a flow-limiting or a differential pressure valve (DPV), which can be of the low, medium or high pressure types. The problem with DPV is that it can cause CSF overdrainage, depending on changes of body position from supine to upright. With the recent introduction of adjustable or programmable valves (see http://www.lifenph.com/), overdrainage and underdrainage can be noninvasively managed by use of a magnetic device.

Another surgical procedure is the endoscopic third ventriculostomy (ETV), whose main indication is in NPH associated with aqueductal stenosis. In an Italian multicenter study of 110 patients operated using ETV for INPH, 76 subjects (69.1%) remained improved after 2 years follow-up, and the complication rate was low,^66^ but the authors caution that their data must be confirmed by further studies.

## Conclusions

In NPH cases, the most important question is not whether the patient has NPH or not, but whether they will benefit from shunt surgery. To tackle this question we have to first base our decision on clinical and tomographic data. To this end, the best clinical predictors of good surgical outcome are short disease duration, high cognitive scores (mild or no dementia), gait disturbance preceding mental deterioration, dilation of temporal horns, and small sulci.^12,13,25,51,57^ Nevertheless, the positive predictive value of the clinical-tomographic data alone is no greater than 65%,^25^ and therefore must be improved upon by using complementary prognostic tests. As regards radionuclide cisternography, ICP monitoring and lumbar infusion tests, we agree with other authors^3,56^ that none of these tests can confirm whether the patient will benefit from surgery, although they are able to show malfunctioning of CSF dynamics. CSF-TT is the only test that can temporarily simulate the effect of a definitive shunt. The one tap CSF-TT has high positive predictive value (in our experience, 100%), but has low sensitivity (26–61%). Therefore, when the test returns a negative result it cannot be used to exclude patients from surgery. In such cases, we have to resort to repeated (RTT) or continuous (LED) CSF removal. The most reliable prediction would be achieved if RTT or LED were to prove positive, in addition to the occurrence of B-waves during more than 50% of ICP recording time.
